# Slope of the estimated glomerular filtration rate and its associated factors among individuals with chronic kidney disease in the general Japanese population

**DOI:** 10.1007/s10157-024-02466-x

**Published:** 2024-02-10

**Authors:** Jun Ito, Masafumi Fukagawa

**Affiliations:** 1 Faculty of Nursing, Hyogo University, 2301, Hiraokacho-Shinzaike, Kakogawa, Hyogo 675-0195 Japan; 2 Division of Nephrology, School of Medicine, International University of Health and Welfare, 4-3, Kozunomori, Narita, Chiba 286-8686 Japan; 3 Division of Nephrology, Endocrinology and Metabolism, School of Medicine, Tokai University, 143, Shimokasuya, Isehara, Kanagawa 259-1193 Japan

**Keywords:** Chronic kidney disease, eGFR slope, Risk factor, Proteinuria, Health checkup, Japanese

## Abstract

**Background:**

To suppress the incidence of end-stage kidney disease, we need to identify chronic kidney disease (CKD) patients with a high risk of rapid decline in the estimated glomerular filtration rate (eGFR). However, the current status of eGFR slope and its associated factors in the Japanese population have not been fully elucidated.

**Methods:**

Among examinees aged 40–70 years in the 2014 Specific Health Checkup conducted by the National Health Insurance in Kobe, Japan (*n* = 61,985), we prospectively observed 7291 examinees with CKD stage G3 from 2014 to 2018.

**Results:**

Until 2018, 4221 examinees continued to undergo annual SHCs for a total of five checkups per subject and had available records of all necessary data. The median eGFR change was −0.22 ml/min/1.73 m^2^/year. Only 9.2% of those subjects showed rapid eGFR decline (faster than −2.0 ml/min/1.73 m^2^/year). Logistic regression analysis identified diabetes, smoking habits, high urinary protein levels, older age, high systolic blood pressure, and low serum low-density lipoprotein cholesterol levels as independent predictors for rapid eGFR decline. Hemoglobin A1c levels did not contribute to the eGFR slope in CKD stage-G3 subjects with diabetes and proteinuria.

**Conclusion:**

Most Japanese CKD stage-G3 subjects had a very slow decline in eGFR. A small proportion of CKD individuals who have a predictive factor of rapid eGFR decline should receive considerable attention from a nephrologist.

## Introduction

Chronic kidney disease (CKD) affects 14.8 million individuals in Japan [1]. Although the number of incident dialysis patients has decreased, the prevalent number of dialysis patients continues to increase by approximately 2000 every year in the country; thus, measures to prevent CKD progression are urgently needed. However, it is not feasible for a few nephrologists to manage such a large number of CKD patients. In fact, there are many patients with a very slow decline in the estimated glomerular filtration rate (eGFR), even in CKD stage G3 or beyond. To suppress the incidence of end-stage kidney disease (ESKD) requiring renal replacement therapies (RRTs) with limited medical resources, it is necessary to explore the predictors of rapidly progressive CKD to determine what types of CKD patients should receive the most attention.

In Japan, all citizens are covered by health insurance. Since 2008, medical insurers have been obliged to provide the Specific Health Checkup (SHC) focusing on metabolic syndrome for all their subscribers aged 40–74 years. This program plays an important role in that it not only provides healthcare guidance based on individual results but also improves prognoses in patients with lifestyle-related diseases by analyzing a large population dataset.

As an indicator of kidney disease progression, eGFR slope is useful. The slope not only serves as a surrogate marker for the development of ESKD, but also is widely used in daily clinical practice such as predicting kidney prognoses, planning treatment strategies, evaluating treatment effects, and patient education. In this study, we revealed the eGFR slope in each clinical status in CKD stage-G3 subjects of Japanese by analyzing the SHC dataset.

## Materials and methods

### Study cohort

**Fig. 1:**
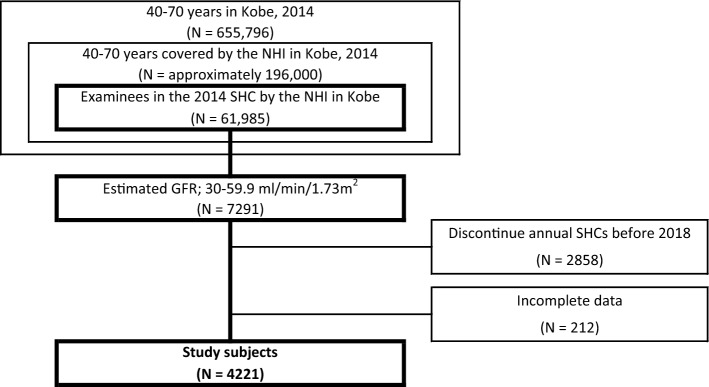
Study subjects. *NHI* national health insurance, *SHC* specific health checkup, *GFR* glomerular filtration rate

Of the 655,796 inhabitants aged 40 to 70 in Kobe City, Japan in 2014, approximately 196,000 had been covered by National Health Insurance (NHI), and 61,985 individuals underwent the 2014 NHI-conducted SHC in Kobe. The checkup rate was 31.6%. Of those 61,985 examinees, we prospectively observed the annual SHC results of 7291 examinees with CKD stage G3, from 2014 to 2018 (Fig. 1).

### Clinical indicators

The data obtained from SHCs, such as systolic blood pressure (SBP), serum low-density lipoprotein cholesterol (sLDL-C), serum uric acid (sUA), hemoglobin A1c (HbA1c) levels, body mass index (BMI), and urinary protein measured by the dip-stick method, were used for each analysis as clinical indicators. Diabetes was defined as HbA1c ≥ 6.5% or use of antidiabetic drugs. A smoking habit was defined as smoking within six months before the start of the observation.

### Evaluation of kidney function

The serum creatinine (sCr) value was measured by the enzymatic method and recorded to two decimal places in the SHC. We calculated an eGFR value for each examinee using the Japanese equation for eGFR from sCr as follows [2]:

$${\text{eGFR}}\left( {{\text{ml}}/\min /1.73\,{\text{m}}^{2} } \right) = 194 \times {\text{sCr}}\left( {{\text{mg}}/{\text{dl}}} \right)^{ - 1.094} \times {\text{age}}\left( {{\text{year}}} \right)^{ - 0.287} \left( {{\text{if}}\,{\text{female}}: \times 0.739} \right)$$

We calculated the mean annual change in eGFR (ΔeGFR; ml/min/1.73 m^2^/year) based on the slope of the regression line of eGFR values from five consecutive annual SHCs.

For the estimation of kidney prognosis, we calculated the predicted eGFR at the end of life (predicted end eGFR) for each subject as follows:

$${\text{Predicted}}\,{\text{end}}\,{\text{eGFR}}\left( {{\text{ml}}/\min /1.73\,{\text{m}}^{2} } \right) = {\text{baseline}}\,{\text{eGFR}}\left( {{\text{ml}}/\min /1.73\,{\text{m}}^{2} } \right) + \left\{ {{\text{life}}\,{\text{expectancy}} - {\text{age}}} \right\}\,\left( {{\text{years}}} \right) \times \Delta {\text{eGFR}}\left( {{\text{ml}}/\min /1.73\,{\text{m}}^{2} /{\text{year}}} \right)$$

The mean life expectancy of Japanese individuals was 81.3 years for males and 87.3 years for females in 2018. Since the average eGFR value at the time of dialysis initiation in Japan was 5.43 ml/min/1.73 m^2^ [3], we defined the subjects whose predicted end eGFR values would be <5 ml/min/1.73 m^2^ as “RRT high-risk subjects” in this study.

In our preliminary analysis in this study, RRT high-risk subjects were divided into groups of 1 ml/min/1.73 m^2^/year of ΔeGFR for screening. {sensitivity-(1-specificity)} was at its maximum (sensitivity 92.9%, specificity 94.6%, odds ratio 229.1, 95% CI 128.3–409.4) when −2 ml/min/1.73 m^2^/year of ΔeGFR was set as the cutoff value. Therefore, we defined a subject whose ΔeGFR was at that level or faster as a “rapid GFR decliner (RGD)” in this study.

### Statistical analyses

To compare the ΔeGFR distribution according to clinical status, the Mann–Whitney U test for comparisons between two groups and the Kruskal–Wallis test among three or more groups were performed. In diabetes subjects with CKD stage G3 and proteinuria, to explore independent factors that contributed to ΔeGFR, linear regression analysis was performed. In addition, we identified RRT high-risk subjects and RGDs among all CKD stage-G3 subjects. To explore independent predictors of RGD among the clinical indicators measured in SHCs, logistic regression analysis was performed. All statistical analyses were performed using IBM SPSS Statistics 26 (IBM Corp., USA). In each analysis, a *P* value <0.05 indicated statistical significance.

## Results

**Fig. 2:**
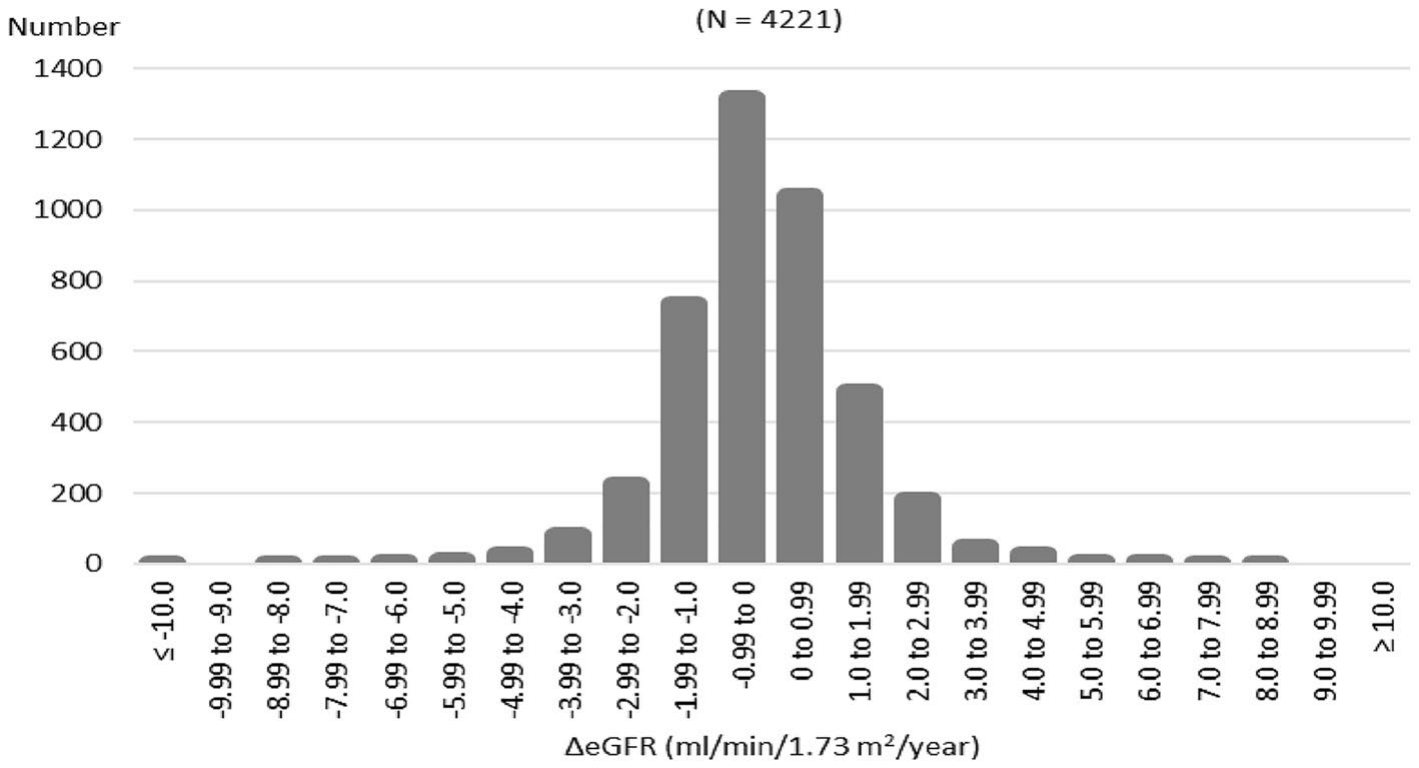
Distribution of ΔeGFR. *ΔeGFR* mean annual change in estimated glomerular filtration rate

**Fig. 3:**
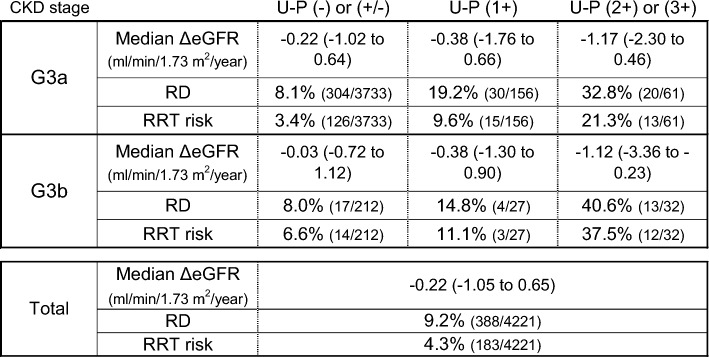
Median ΔeGFR, rapid GFR decliners, and renal replacement therapy high-risk subjects in each CKD stage. *ΔeGFR* mean annual change in estimated glomerular filtration rate, *GFR* glomerular filtration rate, *CKD* chronic kidney disease, *U-P* urinary protein, *RD* rapid glomerular filtration rate decliner, *RRT risk* renal replacement therapy high-risk subject ΔeGFR are indicated as median values and interquartile ranges

Of 7291 examinees with CKD stage G3 in the 2014 SHC in Kobe, 4221 examinees had continued to undergo annual SHCs until 2018, for a total of five checkups per examinee, and had available records of all necessary data (Fig. 1). The baseline characteristics of the 4221 subjects with stage G3 CKD are described in Table 1. The distribution of ΔeGFR values is shown in Fig. 2. The overall median ΔeGFR was −0.22 ml/min/1.73 m^2^/year. During the study period, seven subjects progressed to CKD stage G5, but none of them reached an eGFR of <5 ml/min/1.73 m^2^. In the 4221 subjects, the proportions of RGDs and RRT high-risk subjects were 9.2 and 4.3%, respectively. Figure 3 shows the median ΔeGFR, the proportion of RGDs, and the proportion of RRT high-risk subjects stratified by CKD stages. The distribution of ΔeGFR in each clinical status is shown in Table 2. In males, age, baseline eGFR, baseline SBP, and baseline urinary protein levels were significantly correlated with ΔeGFR. In females, baseline urinary protein levels and a smoking habit were significantly correlated with ΔeGFR. Although no significant difference in the ΔeGFR distribution was found in the overall comparison of subjects with and without diabetes, when limited to subjects with proteinuria, the decline in eGFR was significantly faster in diabetic subjects than in nondiabetic subjects (Table 3). In 69 diabetic subjects with CKD stage G3 and proteinuria, none of the factors, including HbA1c, were associated with ΔeGFR in the linear regression analysis (Table 4).

**Table 1:** Baseline characteristics of study subjects (*n* = 4221)

	Median	(Interquartile range)
Age (year)	67.0	(65.0–69.0)
Sex (male)	47.8%	
BMI (kg/m^2^)	22.7	(20.9–24.7)
eGFR (ml/min/1.73 m^2^)	55.6	(51.9–58.2)
	Stage G3a 93.6%, G3b 6.4%	
Urinary protein	(−) 87.8%, (±) 5.6%, (1+) 4.3%, (2+) 1.7%, (3+) 0.5%	
SBP (mmHg)	128.0	(116.0–138.0)
DBP (mmHg)	76.0	(69.0–83.0)
sLDL-C (mg/dl)	126.0	(106.0–148.0)
sTG (mg/dl)	102.0	(74.0–143.0)
sUA (mg/dl)	5.7	(4.9–6.7)

	Prevalence	(Patients undergoing treatment)
Hypertension	49.2%	(75.9%)
Dyslipidemia	68.2%	(43.8%)
Diabetes	10.6%	(65.9%)
Hyperuricemia	18.9%	
Smoking habit	7.6%	
Subjects who regularly visited a medical institution	51.5%	

Hypertension was defined as SBP ≥ 140 mmHg, DBP ≥ 90 mmHg, or the use of an antihypertensive agent. Dyslipidemia was defined as serum LDL-C level ≥140 mg/dl, serum TG level ≥150 mg/dl, or the use of lipid-lowering medication. Diabetes was defined as hemoglobin A1c level ≥6.5% or the use of an antidiabetic drug. Hyperuricemia was defined as serum UA level ≥7.0 mg/dl

*BMI* body mass index, *eGFR* estimated glomerular filtration rate, *SBP* systolic blood pressure, *DBP* diastolic blood pressure, *sLDL-C* serum low-density lipoprotein cholesterol, *sTG* serum triglyceride, *sUA* serum uric acid

**Table 2:** Median ΔeGFR in each stratified group of baseline clinical data (*n* = 4221)

	Male				Female			
	Number	Median ΔeGFR (ml/min/1.73 m^2^/year)	Interquartile range	*P* value	Number	Median ΔeGFR (ml/min/1.73 m^2^/year)	Interquartile range	*P* value
Total	2016	−0.30	−1.12 to 0.63		2205	−0.15	−0.96 to 0.65	0.01*
Age (year)				<0.001*				0.35
70	442	−0.33	−1.21 to 0.48		381	−0.16	−1.18 to 0.69	
60–69	1492	−0.31	−1.12 to 0.66		1672	−0.15	−0.95 to 0.63	
50–59	65	0.47	−0.53 to 1.53		144	−0.19	−0.81 to 0.90	
40–49	17	−0.61	−1.20 to 0.10		8	0.64	−1.20 to 1.83	
Baseline eGFR (ml/min/1.73 m^2^)				0.002*				0.07
59–50	1577	−0.36	−1.18 to 0.59		1910	−0.18	−0.97 to 0.64	
49–40	373	−0.05	−0.90 to 0.90		257	0.01	−0.84 to 0.87	
39–30	66	−0.25	−1.03 to 0.85		38	−0.33	−1.43 to 0.82	
Baseline BMI (kg/m^2^)				0.33				0.11
30–	50	−0.34	−1.16 to 0.97		54	−0.11	−1.05 to 0.81	
25–29.9	536	−0.17	−1.07 to 0.74		314	−0.02	−0.83 to 0.78	
18.5–24.9	1406	−0.32	−1.15 to 0.56		1629	−0.17	−0.97 to 0.65	
−18.4	24	−0.36	−0.90 to 1.29		208	−0.32	−1.05 to 0.49	
Baseline SBP (mmHg)				<0.001*				0.81
160–	89	−0.64	−1.51 to 0.50		64	−0.35	−1.28 to 0.66	
140–159	407	−0.46	−1.29 to 0.47		321	−0.28	−1.08 to 0.65	
120–139	974	−0.28	−1.07 to 0.56		1034	−0.16	−0.94 to 0.62	
100–119	504	−0.15	−0.90 to 0.86		661	−0.12	−0.95 to 0.68	
−99	42	−0.53	−1.29 to 0.72		125	−0.09	−0.97 to 0.81	
Baseline sLDL-C (mg/dl)				0.48				0.73
160–	218	−0.23	−0.98 to 0.65		420	−0.13	−0.90 to 0.66	
140–159	363	−0.29	−1.08 to 0.66		420	−0.17	−1.02 to 0.61	
120–139	478	−0.30	−1.04 to 0.53		558	−0.22	−1.06 to 0.66	
100–119	503	−0.36	−1.11 to 0.52		476	−0.11	−0.92 to 0.61	
−99	454	−0.27	−1.31 to 0.90		331	−0.14	−0.91 to 0.79	
Baseline sUA (mg/dl)				0.26				0.96
8.0–	211	−0.30	−1.15 to 0.93		33	−0.04	−1.71 to 1.08	
7.0–7.9	439	−0.24	−1.06 to 0.67		114	−0.25	−1.13 to 0.85	
6.0–6.9	683	−0.37	−1.12 to 0.48		358	−0.14	−1.10 to 0.67	
−5.9	683	−0.27	−1.12 to 0.65		1700	−0.15	−0.94 to 0.64	
Urinary protein				0.001*				<0.001*
3+	16	−1.05	−4.03 to 0.30		5	−2.04	−4.43 to −1.39	
2+	55	−1.03	−2.28 to 0.58		17	−1.51	−2.82 to −0.03	
1+	122	−0.49	−1.75 to 0.67		61	−0.21	−1.28 to 0.88	
− or ±	1823	−0.28	−1.07 to 0.64		2122	−0.14	−0.95 to 0.66	
Diabetes				0.11				0.48
With diabetes	305	−0.36	−1.47 to 0.83		144	−0.21	−1.50 to 0.90	
Without diabetes	1711	−0.28	−1.08 to 0.62		2061	−0.15	−0.95 to 0.65	
Smoking habit				0.44				0.02*
Smoking	255	−0.31	−1.37 to 0.66		65	−0.55	−1.60 to 0.46	
Not smoking	1761	−0.30	−1.09 to 0.63		2140	−0.15	−0.95 to 0.65	

*ΔeGFR* mean annual change in estimated glomerular filtration rate, *eGFR* estimated glomerular filtration rate, *BMI* body mass index, *SBP* systolic blood pressure, *sLDL-C* serum low-density lipoprotein cholesterol, *sUA* serum uric acid

^*^P < 0.05

**Table 3:** Comparison of ΔeGFR with and without diabetes and urinary protein (*n* = 4221)

	U-P ≤±			U-P ≥ 1+			
	Median ΔeGFR (ml/min/1.73 m^2^/year)	Interquartile range	Number	Median ΔeGFR (ml/min/1.73 m^2^/year)	Interquartile	Number range	*P* value
Diabetes	−0.22	−1.20 to 0.99	380	−1.29	−3.18 to 0.16	69	<0.001*
Without diabetes	−0.20	−0.98 to 0.63	3565	−0.40	−1.65 to 0.65	207	0.02*
*P* value	0.86			0.001*			

*ΔeGFR* mean annual change in estimated glomerular filtration rate, *U-P* urinary protein

^*^P < 0.05

**Table 4:** Contributing factors to ΔeGFR in diabetic subjects with CKD stage G3 and proteinuria (*n* = 69)

	*Β*	*t*	*P* value
Age	−0.11	−0.85	0.40
Sex (Male)	0.05	0.38	0.71
Baseline eGFR	−0.04	−0.34	0.74
Baseline SBP	−0.09	−0.66	0.51
Baseline HbA1c	0.17	1.32	0.19

*ΔeGFR* mean annual change in estimated glomerular filtration rate, *CKD* chronic kidney disease, *eGFR* estimated glomerular filtration rate, *SBP* systolic blood pressure, *HbA1c* hemoglobin A1c

A logistic regression analysis on the proportion of RGDs showed that diabetes (*P* < 0.001, odds ratio: OR 2.15), smoking habits (*P* = 0.002, OR 1.72), a higher baseline urinary protein level (*P* < 0.001, OR 1.58 per step), older age (*P* = 0.01, OR 1.51 per 10 years), a higher baseline SBP (*P* = 0.008, OR 1.09 per 10 mmHg), and a lower baseline sLDL-C level (*P* = 0.01, OR 1.04 per 10 mg/dl) were independent predictors (Table 5).

**Table 5:** Association between clinical indicators and the proportion of rapid GFR decliners by the logistic regression analysis (*n* = 4221)

		*P* value	Odds ratio	95% confidential interval
Sex	(male)	0.28	0.87	0.68–1.12
Age	(per 10 years)	0.01*	1.51	1.10–2.09
Estimated GFR	(per 10 ml/min/1.73 m^2^)	0.73	1.04	0.85–1.26
BMI	(per 1 kg/m^2^)	0.18	0.98	0.94–1.01
SBP	(per 10 mmHg)	0.008*	1.09	1.02–1.16
sLDL-C	(per 10 mg/dl)	0.01*	0.96	0.92–0.99
sUA	(per 1 mg/dl)	0.051	1.10	1.00–1.21
Urinary protein	(per each step)	<0.001*	1.58	1.40–1.78
Diabetes	(yes vs. no)	<0.001*	2.15	1.62–2.86
Smoking habit	(yes vs. no)	0.002*	1.72	1.22–2.43

*GFR* glomerular filtration rate, *BMI* body mass index, *SBP* systolic blood pressure, *sLDL-C* serum low-density lipoprotein cholesterol, *sUA* serum uric acid

^*^P < 0.05

## Discussion

We used ΔeGFR as an indicator of changes in kidney function because it can be utilized easily in a wide range of daily clinical practices as mentioned in the introduction section. The noteworthy point of the present study is that the decline rate in eGFR was remarkably slower than previous reports. Since the early 2000s, various epidemiological studies on CKD have been reported. In Norway, the mean ΔeGFR, determined using a hospital database, for patients with CKD stage G3 was −1.03 ml/min/1.73 m^2^/year [4]. In elderly individuals with CKD stage G3 in Canada, the mean ΔeGFRs were −3.6 and −2.8 mL/min/1.73 m^2^/year in males and females with diabetes, respectively, and −1.9 and −1.1 mL/min/1.73 m^2^/year in males and females without diabetes, respectively [5]. In a previous study in the general Japanese population, using a database of health checkups of the two periods over 10 years, 1988–1993 and 1998–2003, the mean ΔeGFR was as slow as −0.36 ml/min/1.73 m^2^/year [6]. It is not clear why eGFR declines slower in Japanese CKD patients than in Western CKD patients. However, some differences in background factors of Japanese CKD patients compared to Western CKD patients has been reported, such as low complication rate of arteriosclerotic disease, low BMI, low serum CRP levels [7, 8]. It is speculated that those factors may have affected the differences in eGFR slope between races. In our results, ΔeGFR (median; −0.22 ml/min/1.73 m^2^/year, mean; −0.19 ml/min/1.73 m^2^/year) in CKD stage-G3 subjects was even slower than the result of similar study previously conducted in Japan using health checkup data [6]. Since 2002, awareness of CKD has increased, and total CKD management has become widespread. The difference in the results between the previous report and the present study may reflect the advances over the last two decades. In addition, all the subjects in this study underwent the SHC every year, and those who met the criteria for metabolic syndrome had opportunities to receive healthcare guidance through a public health nurse. The very slow decline in eGFR across this population may have resulted from the program.

The Kidney Disease Improving Global Outcomes (KDIGO) 2012 Clinical Practice Guideline for the Evaluation and Management of CKD defined rapid progression as a sustained decline in eGFR of 5 ml/min/1.73 m^2^/year or more [9]. However, few studies are available regarding the optimal definition of rapid progression [9]. According to an analysis of 529,312 individuals using the Alberta Kidney Disease Network database cited in the 2012 KDIGO guideline, the risk of ESKD increased continuously by approximately twofold for every 1 ml/min/1.73 m^2^/year decrease in ΔeGFR, and the basis for the cutoff value of 5 ml/min/1.73 m^2^/year is not clear. When ΔeGFR < -2 ml/min/1.73 m^2^/year, the risk of ESKD was shown to be 2.71 times (2.08–3.53) compared to a group with stable kidney function [9], but a large number of individuals with eGFR ≥ 60 ml/min/1.73 m^2^ had been included in the study subjects. The risk is therefore presumed to be even higher in individuals with CKD stage G3. In the present study, we determined the cutoff value for RGD as ΔeGFR < −2 ml/min/1.73 m^2^/year, as we mentioned in the materials and methods section.

In addition, we also attempted to analyze according to the KDIGO’s definition of rapid progression, but the analysis could not be performed because only 28 of the 4221 subjects (0.66%) had ΔeGFR < −5 ml/min/1.73 m^2^/year. While in a cohort study in the UK, it has been reported that 24% of CKD cases aged 60 and over had a ΔeGFR < −5 ml/min/1.73 m^2^/year [10], in the present study, there were extremely few subjects with ΔeGFR < −5 ml/min/1.73 m^2^/year. Considering that the total number of ESKD patients is nevertheless increasing in Japan, for CKD stage G3 of Japanese, it may be insufficient to focus only on individuals with ΔeGFR < −5 ml/min/1.73 m^2^/year.

In unadjusted analyses, a baseline urinary protein level was significantly correlated with ΔeGFR in both sexes. Then, among nondiabetic subjects, eGFR declined twice as fast in subjects with proteinuria compared with those without proteinuria, and among diabetic subjects, those with proteinuria had a fivefold faster decline in eGFR than those without proteinuria. In the logistic regression analysis, diabetes and urinary protein levels were significantly associated with the proportion of RGDs irrespective of baseline eGFR, and we consider those factors to be strong predictors of rapid GFR decline, as has been previously reported [5–7]. In diabetic subjects with CKD stage G3 and proteinuria, HbA1c levels did not contribute to ΔeGFR. To date, it has been reported that HbA1c levels are associated with the development of albuminuria, and that glycemic control suppresses progression to the microalbuminuria or overt proteinuria phase and reverses from the microalbuminuria phase to the normal-urine phase [11–14]. However, there is not enough evidence that the eGFR slope is improved by strict blood glucose control after the overt proteinuria phase or once kidney function begins to decrease. Our results are consistent with those previous findings.

A large-scale observation showed that smoking was a risk factor for the progression of CKD [15]. Also, in our study population, having a smoking habit was identified as an independent predictor of RGD. However, in this study, there were no available data on the number of cigarettes smoked, duration of smoking, and smoking history. More detailed data are needed to determine the association between smoking habits and ΔeGFR and the effect of smoking cessation on improving the eGFR slope.

In unadjusted analyses, a higher baseline SBP was significantly correlated with a faster decline in eGFR in males. While, the correlation between those two variables did not show a statistically significant difference in females. The results may have been influenced by the fact that a large proportion of hypertensive subjects in our study population had already taken antihypertensive drugs, and only 2.9 and 1.5% of female subjects had a baseline SBP and a mean SBP of 160 mmHg or higher, respectively. In the logistic regression analysis, a high baseline SBP was significantly associated with the risk of RGD. Thus, there is no doubt that high BP is an important factor that exacerbates CKD, as previously reported [7, 16, 17].

A lower baseline sLDL-C level was associated with a higher risk of RGD in our study population. The result was inconsistent with the findings of a meta-analysis that showed that high sLDL-C was associated with a high risk of CKD [18]. However, in our study, the statistical significance disappeared in an analysis excluding the subjects with baseline sLDL-C levels below 70 mg/dl (*n* = 96, median ΔeGFR; −0.58 ml/min/1.73 m^2^/year). Given the above, we speculated not that a higher sLDL-C level was preferable but rather that underlying factors of an extremely low sLDL-C level, such as undernutrition or chronic inflammation, may have contributed to the rapid decline in eGFR. To verify this hypothesis, further investigation, including evaluation of nutritional status, serum albumin, and serum C-reactive protein, is needed. In addition, in our study population, the lower the sLDL-C-level group, the higher the rate of taking a lipid-lowering drug (Pearson’s *χ*^2^ test; *p* < 0.001), suggesting that past histories of high sLDL-C levels may have affected the study results.

The strength of the present study is that the ΔeGFR values are considered high confidence because those were determined using the slopes of the regression lines of the eGFR values of five consecutive times at 1-year intervals over four years, for 4221 subjects. The target area, Kobe City, includes a range of environments from urban to rural. The population per nephrologist of approximately 24,000 was almost the same as the national average (approximately 25,000), and the number of chronic dialysis patients per million people covered by the NHI in Kobe City in 2018 was 2673, which was nearly equal to the national average of 2688. It is presumed that the situation of medical care for CKD in the target area of this study is not remarkably different from the situation in Japan as a whole.

However, the participation rate for the 2014 SHC in Kobe was 31.6%, and thus, the existence of selection bias cannot be denied, which is the biggest limitation in this study. Moreover, it is impossible to follow the examinees who had discontinued SHC participation during the observation period.

There were also no urinary albumin quantification data, and we used a dipstick urine test to evaluate proteinuria. It was reported that urinary protein by a dipstick test of ± or above had a high specificity (95.6%) but low sensitivity (66.2%) and low positive predictive value (17.5%) to detect CKD stage A2 or higher [19]; thus, its inferior accuracy is also a limitation.

The rate of GFR decline may be relatively constant over time in an individual [9, 20]. However, there is no evidence that eGFR slope accurately predicts the time to ESKD. It was reported that the longer the follow-up time, the higher the probability of non-linear trajectory of eGFR decline [9, 21]. The causes of nonlinearity include not only external factors such as intercurrent illness and changes in medication, but also intrinsic to the disease process [21]. Although the present study used data over a relatively long period of time, the accuracy of very long-term predictions on eGFR at the end of life requires further verification.

It is an important issue how many subjects who had transitioned from acute kidney injury (AKI) to CKD among RGDs in the present study. However, in the present study, there was no information on the history of AKI, and only the data at 1-year intervals were available. Thus, it was difficult to determine whether the subjects had developed AKI.

The use of renin-angiotensin system (RAS) inhibitors and sodium glucose co-transporter 2 (SGLT2) inhibitors greatly affects the eGFR slope in CKD patients. However, the SHC dataset used in this study only contained information on the overall use of antihypertensive and antidiabetic drugs, and information on the use of RAS inhibitors and SGLT2 inhibitors was not included. In this study population, it is considered that a substantial number of subjects had been prescribed RAS inhibitors. Although the kidney protective effect of SGLT2 inhibitors in nondiabetic CKD patients had not been revealed until 2020 [22], the use of SGLT2 inhibitors had already been widespread in diabetic patients during the observation period (from 2014 to 2018). Therefore, it is speculated that our results were affected by SGLT2 inhibitors in some subjects with diabetes. The lack of information regarding RAS inhibitors and SGLT2 inhibitors is an important limitation of the present study.

## Conclusion

Among subjects with CKD stage G3 in Japan, the decline rate in eGFR (median 0.22 ml/min/1.73 m^2^/year) was remarkably slower than the result of similar study previously conducted in Japan. Only 9.2% of study subjects had ΔeGFR < −2 ml/min/1.73 m^2^/year. These results may reflect the advances of countermeasures against CKD in Japan. In nondiabetic subjects, eGFR declined twice as fast in those with proteinuria compared with those without proteinuria, and in diabetic subjects, those with proteinuria had a fivefold faster decline in eGFR than those without proteinuria. In diabetic subjects with CKD stage G3 and proteinuria, HbA1c levels did not contribute to ΔeGFR. A logistic regression analysis revealed that independent predictors for RGD were diabetes, smoking habits, proteinuria, older age, and hypertension, as previously reported. Regarding the significance of a low sLDL-C level for RGD, further investigation is required. To efficiently suppress the number of incident ESKD patients with limited medical resources, we consider it important to preferentially refer those with predictors to a nephrologist.

## Data Availability

The raw data analyzed during the present study are not publicly available according to the ethical approval and contract with Kobe City. If anyone has questions about the data, please contact the author.
